# Exome sequencing in extreme altitude mountaineers identifies pathogenic variants in *RTEL1* and *COL6A1* previously associated with respiratory failure

**DOI:** 10.14814/phy2.16015

**Published:** 2024-04-23

**Authors:** Evgeniia M. Maksiutenko, Valeriia Merkureva, Yury A. Barbitoff, Victoria V. Tsay, Mikhail V. Aseev, Andrey S. Glotov, Oleg S. Glotov

**Affiliations:** ^1^ Department of Genomic Medicine D.O. Ott Research Institute of Obstetrics, Gynaecology, and Reproductology St. Petersburg Russia; ^2^ CerbaLab Ltd. St. Petersburg Russia; ^3^ Department of Genetics and Biotechnology St. Petersburg State University St. Petersburg Russia; ^4^ Department of Experimental Medical Virology Molecular Genetics and Biobanking of Pediatric Research and Clinical Center for Infectious Diseases St. Petersburg Russia

**Keywords:** exome sequencing, high altitude adaptation, hypoxia, mountaineering, pathogenic variant

## Abstract

Adaptation of humans to challenging environmental conditions, such as extreme temperature, malnutrition, or hypoxia, is an interesting phenomenon for both basic and applied research. Identification of the genetic factors contributing to human adaptation to these conditions enhances our understanding of the underlying molecular and physiological mechanisms. In our study, we analyzed the exomes of 22 high altitude mountaineers to uncover genetic variants contributing to hypoxic adaptation. To our surprise, we identified two putative loss‐of‐function variants, rs1385101139 in *RTEL1* and rs1002726737 in *COL6A1* in two extremely high altitude (personal record of more than 8500 m) professional climbers. Both variants can be interpreted as pathogenic according to medical geneticists' guidelines, and are linked to inherited conditions involving respiratory failure (late‐onset pulmonary fibrosis and severe Ullrich muscular dystrophy for rs1385101139 and rs1002726737, respectively). Our results suggest that a loss of gene function may act as an important factor of human adaptation, which is corroborated by previous reports in other human subjects.

## 1 INTRODUCTION

Identification of the genetic factors that confer adaptation to environmental stresses, such as extreme temperature, malnutrition, or hypoxia, is a very important step towards understanding human biology and enhancing stress resistance. High altitude adaptation is of particular interest for researchers as it provides a good model for investigating genetic and physiological determinants of hypoxic response and resistance to hypoxia.

Recent genome‐wide analyses of the highland populations led to detection of a large amount of genomic changes responsible for high altitude adaptation. The majority of these genetic variants associated with adaptation to hypoxia were found among Tibetan, Andean, and Ethiopian individuals, and many such variants seem to be unique to each population (Simonson, 2015). In most cases adaptive genetic changes affect hypoxia‐inducible factor (HIF) pathway genes; this pathway mediates the transcriptional response to hypoxia by transducing changes in cellular O_2_ levels to changes in gene expression. Predominantly such studies focus on variants which are located in *EGLN1* and *EPAS1* genes—key components of the HIF pathway, and peroxisome proliferator‐activated receptor α (*PPARA*), which is down‐regulated by HIF and play a role of transcriptional regulator of fatty acid oxidation in liver, heart, and muscle (Horscroft et al., 2017; Storz & Cheviron, 2021).

Furthermore, several studies investigated changes in transcriptional gene regulation on extreme altitudes. One of such studies showed daily variation in expression, as well as in altitude‐dependent expression pattern, of immune‐related genes upon an expedition to the highest city in the world, La Rinconada, Peru (5100 m) (Manella et al., 2022). Another study revealed that increased expression of an important regulator in stem cells, *OCT4*, at extreme altitude can directly elevate the expression of hemoglobin genes (Chen et al., 2012).

Despite the aforementioned progress in the field, the genomes of professional mountaineers capable of climbing to extreme heights may provide additional insights into the genetic changes that confer resistance to hypoxia and altitude sickness. In our study, we applied exome sequencing to 22 mountaineers, in order to discover the rare variants that may contribute to their high‐altitude hypoxia adaptation. Surprisingly, our analysis revealed two subjects carrying pathogenic variants associated with inherited conditions linked to respiratory failure.

## 2 MATERIALS AND METHODS

### 2.1 Description of donors

The analysis included 22 (mean age 50.5, 82% [*n* = 18] male, 18% [*n* = 4] female) mountaineers and 499 control samples without early‐onset monogenic disorders. The set of mountaineers included professional mountain guides who regularly accompany people to 7000+ m, as well as high‐altitude climbers with 8000+ m experience. The group also includes those who participated in high‐altitude ascents as a part of the Russian national team.

The set of controls was selected from the RUSeq database (Barbitoff et al., 2021), and mainly comprised samples used in a recent association analysis for COVID‐19 (Shcherbak et al., 2022). The inclusion of controls was based on clear indication of health status, same sequencing technology and library preparation method, age (only donors aged 70 years and younger were included) (mean age = 42.4 years, 54% female, 46% male). Donors who experienced severe forms or died from COVID‐19 were also excluded.

### 2.2 Ethics statement

The study was approved by the Ethics Committee of the D.O. Ott Research Institute of Obstetrics, Gynecology, and Reproductology, protocol number 117 dated 19.04.2022. All study participants provided their written informed consent.

### 2.3 DNA library preparation and sequencing

DNA for sequencing was extracted from peripheral blood samples. Library preparation was performed using the Roche Inherited Disease Panel (IDP) v2 clinical exome sequencing kit and KAPA HyperExome kit in accordance with the manufacturer's protocol (https://rochesequencingstore.com/catalog/kapa‐hyperexome/). Libraries were sequenced using Illumina HiSeq 2500/4000, Illumina NovaSeq 6000, or DNBSEQ G400 sequencing platforms.

### 2.4 Bioinformatic analysis

Exome sequencing data analysis was performed using the standard cohort genotyping pipeline. Reads were aligned onto the GRCh38 human reference genome assembly using the bwa‐mem2 tool (Vasimuddin et al., 2019), Alignment results were processed using the Genome Analysis Toolkit (McKenna et al., 2010; Van der Auwera et al., 2013) v. 4.1.9.0. The preprocessing included marking of duplicate reads and base quality score recalibration. After this step, variant calling was performed using GATK HaplotypeCaller in the GVCF mode followed by joint genotyping of all samples. Variants were filtered using the variant quality score recalibration method (DePristo et al., 2011). Genotypes with a total depth of less than 10 reads were set to missing. Finally, variant annotation was performed using the Ensembl VEP toolkit.

After generation of the final variant call set, the dataset was analyzed using the Hail framework for Python (https://hail.is/). Samples were filtered using the following criteria: heterozygous‐to‐homozygous variant ratio range (1; 2.2), transition/transversion ratio range (2.35; 2.75), insertion/deletion ratio range (0.75; 0.95), median genotype quality >30, After sample filtering, an additional round of variant filtering was performed, with all multiallelic variant sites and variants with call rate <90% excluded.

The resulting filtered set of samples and variants was converted to table format and interpreted in accordance with the ACMG Standards and Guidelines for variant interpretation (Richards et al., 2015). The Franklin platform (https://franklin.genoox.com/clinical‐db/home) was used to validate interpretation results.

## 3 RESULTS

We started our analysis by performing exome sequencing and genotyping of a set of 22 high‐altitude mountaineers and a set of 499 healthy control samples (see Section 2 for details). After variant‐ and sample‐level filtering, we detected 78,354 high‐quality variants in 520 samples (498 of which were controls and 22 were cases). Of these, 53,657 variants were synonymous or other low‐impact variants, 23,824 were missense substitutions or in‐frame indels, and 873 were putative loss‐of‐function variants, including nonsense variants, frameshift indels, and canonical splice site variants. In a total set of variants, 14,384 were common (minor allele frequency >5%) and 63,970 were rare. 38,269 variants occurred only once in a complete dataset. To select potentially clinically significant variants, we next filtered out low impact and missense variants and those that manifest in healthy controls. This analysis identified 81 variants that were then interpreted according to the ACMG standards and guidelines for variant interpretation. As a result, we discovered two variants which can be classified as pathogenic causal variants for severe inherited diseases (Table 1). Both of these variants were present in elite mountaineers and were absent both from the control subgroup and from other individuals in the RUSeq database.

**TABLE 1:** Summary information about identified pathogenic variants and carrier subjects.

Parameter	Sample AL004	Sample BAL035
Donor information		
Sex	Male	Male
Age	69	69
Altitude record	8516 m (Lhotse)	8848 m (Everest)
Variant information		
Variant location	chr20:63695594C>T (GRCh38) 20:62326947C>T (b37)	chr21:45998172G>A (GRCh38) 21:47418086G>A (b37)
dbSNP ID	rs1385101139	rs1002726737
Gene name	*RTEL1*	*COL6A1*
Gene product	Essential iron–sulfur (FeS) containing DNA helicase	Component of type VI collagen fibril
Variant consequence	Stop gained	Donor splice site variant
HGVS	NM_016434.3:c.3652+114C>T NP_001269938.1:p.Gln1256Ter	NM_001848.3:c.1575+1G>A
gnomAD AF	3.1 × 10^−5^ (v2.1); 6.5 × 10^−6^ (v3.1)	Absent
Diseases associated with the gene	Pulmonary fibrosis (OMIM:616373); idiopathic pulmonary fibrosis^a^	Collagen VI‐related myopathies: a broad spectrum of disorders ranging from severe Ullrich muscular dystrophy (OMIM:254090) to mild Bethlem myopathy (OMIM:158810)

^a^ Gene‐disease association is not described in OMIM.

The first incidental finding is a pathogenic heterozygous variant in the *RTEL1* gene in a 69 years old high‐altitude climber with a personal altitude record of 8516 m. The subject is an alpinist‐technician and participated in a large number of high‐altitude ascents (five peaks, over 7 km each, several difficult technical ascents to 6000+ m peaks). For several years he worked as a high‐altitude guide with tourists. In 2002 he climbed the Lhotse peak (8.5 km) without the help of Sherpas, but using supplementary oxygen. The donor does not experience health issues during climbing at this time. Nowadays, the donor rarely goes to high altitudes and reports frequent acute respiratory infections with damage to the respiratory system.

**FIGURE 1:**
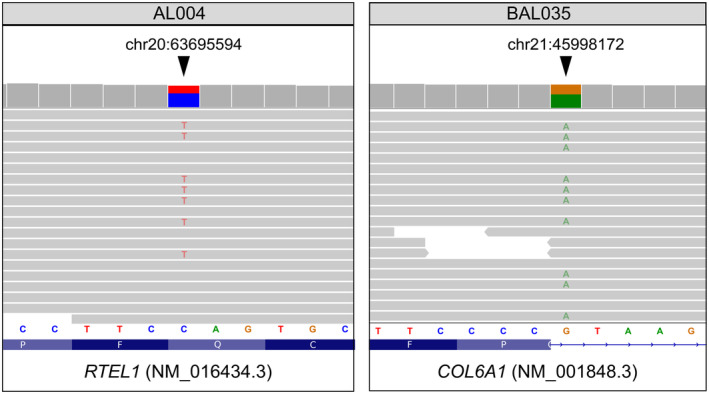
Pathogenic genetic variants identified in the high altitude mountaineers. Shown are read alignments in the variant regions visualized using the Integrated Genomics Viewer (IGV) browser (Robinson et al., 2011). On the left, the rs1385101139 variant in *RTEL1* linked to late‐onset pulmonary fibrosis. On the right, the rs1002726737 variant in *COL6A1* linked to severe Ullrich muscular dystrophy. Coordinates of genetic variants are shown with respect to the GRCh38 human reference genome assembly.

The identified variant, chr20:63695594C>T (rs1385101139) (Figure 1), results in a premature stop codon in the *RTEL1* reading frame. The variant is extremely rare and occurred only once in gnomAD v2.1.1 and gnomAD v3.1.2, with allele frequency 3.1 × 10^−5^ and 6.5 × 10^−6^, respectively. The *RTEL1* gene encodes an essential iron–sulfur (FeS) containing DNA helicase that is crucial for telomere maintenance and DNA repair (Ding et al., 2004). Heterozygous mutations in *RTEL1* were reported to be a major genetic cause of familial pulmonary fibrosis. Pulmonary fibrosis is a general term used to describe a group of fibrosing interstitial lung diseases characterized by accumulation of extracellular matrix and fibroblasts in the distal lung, also may appear as a sporadic disease without extrapulmonary involvement (idiopathic interstitial pneumonia), commonly causes chronic respiratory failure (Marchioni et al., 2018). Idiopathic pulmonary fibrosis is the most frequent (60%) and severe presentation, with a median age at diagnosis of 66 years and median survival of 3–5 years. The most frequent pattern of genetic transmission of pulmonary fibrosis is autosomal dominant (Kannengiesser et al., 2015). In addition to pulmonary fibrosis, it was shown that other *RTEL1* variants (e.g., rs2297440) increase risk of glioma and astrocytoma (Jin et al., 2015). Besides other biallelic mutations in *RTEL1* lead to severe telomere biology disorder called Hoyeraal Hreidarsson syndrome. Cells of *RTEL1*‐deficient patients carrying homozygous and compound heterozygous mutations carry short and dysfunctional telomeres (Jullien et al., 2016).

Another variant was found in another 69‐years‐old male subject from the elite climber group. The donor is an outstanding high‐altitude climber, and participated in several ascents to 7000+ and 8000+ peaks, including Everest (without oxygen support). He had multiple minor and major injuries over the course of his career, and was prescribed celecoxib for a number of years. The donor is currently climbing 7000+ m on occasion.

We found a pathogenic splice donor variant in *COL6A1* chr21:45998172G>A (rs1002726737) which is absent from the gnomAD database. The variant leads to skipping of exon 27 of the gene, as shown previously (Lampe et al., 2008). Loss‐of‐function variants in *COL6A1* result in collagen VI‐related myopathies encompassing a spectrum of disease ranging from severe Ullrich muscular dystrophy to mild Bethlem myopathy (Bönnemann, 2011; Koppolu et al., 2017). Generally, Bethlem myopathy is a slowly progressing muscular dystrophy with onset in infancy that leads to joint contractures and most often affecting the fingers, wrists, elbows, and ankles, sometimes accompanied by progressive respiratory compromise depending on severity of muscle involvement (Haq et al., 1999; Shahrizaila et al., 2006). On the other hand, Ullrich congenital muscular dystrophy is characterized by proximal joint contractures, distal joint hyperlaxity, proximal muscle weakness, scoliosis and respiratory failure (Nadeau et al., 2009; Yonekawa et al., 2013). In both cases respiratory insufficiency usually manifests following the loss of the ability to walk, but some patients can develop impending respiratory insufficiency earlier. Eventually, the combination of weakness and contractures can lead to walking difficulties—about two‐thirds of patients over the age of 50 years require help with ambulation, usually using a mobility scooter or wheelchair (Bönnemann, 2011). These data make the presence of a pathogenic *COL6A1* variant in a middle‐aged high‐altitude climber even more surprising. Notably, the identified rs1002726737 variant has previously been reported as causal for severe Ulrich muscular dystrophy (Lampe et al., 2008) in a homozygous state, but no evidence of its pathogenicity in a heterozygous state has yet been presented. Moreover it was found that *COL6A1* is a hypoxia responsive gene and has been implicated in the Ethiopian pattern of high altitude adaptation (Alkorta‐Aranburu et al., 2012).

## 4 DISCUSSION

In our analysis, we discovered two pathogenic variants associated with respiratory failure‐involving inherited disease. Identification of such variants in extreme altitude mountaineers, both of whom have personal records of climbing to around 8.5 km, poses a question if these variants have a role in driving high altitude adaptation in these individuals.

First, the functional impact of the identified variants has to be considered in more detail. For the rs1002726737 in *COL6A1*, molecular genetic studies have demonstrated the impact of the variant on exon skipping (Lampe et al., 2008). Moreover, the same variant is reported as a likely pathogenic variant in the ClinVar database, while automated interpretation based on ACMG criteria using Franklin classifies the variant as a confidently pathogenic one. At the same time, Lampe et al. (2008) reported two carriers of the heterozygous rs1002726737 variant—parents of a severe UMD patient homozygous for this mutation. These data suggest that, while the variant causes severe disease in a homozygous form, its manifestation in a heterozygote may be much milder. For the rs1385101139 in *RTEL1*, the pathogenic status is also supported by the ACMG criteria. However, heterozygous LoF variants in *RTEL1* results in a very late‐onset pulmonary fibrosis, and the gene itself is under modest evolutionary constraint according to gnomAD data.

The aforementioned details suggest that both rs1385101139 and rs1002726737 variants are not responsible for severe early‐onset disease in a heterozygous state. At the same time, functional evidence suggests that these variants could still affect the function of the corresponding proteins, though milder, late‐onset forms of disease may be driven by a single mutant allele at these loci. It is quite challenging to obtain evidence of the positive impact of rs1385101139 and rs1002726737 variants on high‐altitude adaptation even outside an extreme altitude environment for two main reasons. Firstly, it seems nearly impossible to find a matching control group to distinguish the environmental (i.e., exercise‐driven) effects on fitness from the genetic ones. The proper control individuals should have a similar experience and (at least partially) matching genotype. Secondly, to prove the impact of the identified variants on the phenotype, one would require more individuals bearing such variants to draw statistically supported conclusions. As the variants in question are extremely rare in the population (allele frequency in gnomAD <10^−5^), finding more carrier individuals appears a complicated and lengthy task. Nevertheless detection of these variants in high‐altitude mountaineers adds another example to a series of earlier studies reporting pathogenic alleles in individuals that show resilience to altitude.

For example, investigations of centenarian genomes revealed many Mendelian mutations that allow the individual carriers to achieve exceptional longevity. Freudenberg‐Hua et al. (2014) reported about 130 coding variants that were identified and annotated as “pathogenic” or “likely pathogenic”. All these variants were associated with a wide range of degenerative, neoplastic, and cardiac diseases with autosomal dominant, autosomal recessive, and X‐linked inheritance. Moreover, risk variants for late onset neurodegenerative diseases, such as the *APOE* e4 allele that was even present in a homozygous state in one centenarian who did not develop Alzheimer's disease (Freudenberg‐Hua et al., 2014). In another study, the genome of one supercentenarian had a pathogenic mutation in DSC2, known to predispose to arrhythmogenic right ventricular cardiomyopathy, which is a potentially fatal condition, causing affected individuals to die of sudden cardiac death. Even with this pathogenic mutation, the proband lived to over 110 years (Gierman et al., 2014). Furthermore, earlier works by our group dedicated to the study of longevity also revealed notable genetic associations. In particular, it was noted that the frequency of heterozygous genotypes (carriers of the risk allele) at polymorphic sites in *PPARA*, *PPARD*, *PGC1*, and *UCP3* is higher in individuals aged 69 years or older. This finding may be related to better adaptation of heterozygotes to different environmental conditions (Tarkovskaya et al., 2012).

All these findings indicate that the presence of certain deleterious genetic variants in a person's genotype, can be, at the very least, compatible with greater resistance to environmental stresses, or might even provide certain advantages. These findings are especially important given that more and more people in the world are getting their genome or exome sequenced. In light of these developments, researchers and clinicians will frequently face the question of interpreting the significance of genetic variants with incomplete penetrance (Glotov et al., 2023). Therefore, accumulation of data on the presence of presumably pathogenic variants in healthy individuals (especially in groups of highly skilled athletes, individuals involved in extreme activities, or elderly people) becomes crucial for the introduction of genomic medicine into clinical practice.

## AUTHOR CONTRIBUTIONS

Valeriia Merkureva, Mikhail V. Aseev, Andrey S. Glotov, and Oleg S. Glotov onceived and designed research, Valeriia Merkureva and Victoria V. Tsay performed experiments, Evgeniia M. Maksiutenko and Yury A. Barbitoff analyzed data, all authors interpreted results of experiments, Evgeniia M. Maksiutenko and Yury A. Barbitoff prepared figures, Evgeniia M. Maksiutenko and Yury A. Barbitoff drafted manuscript, all authors edited and revised manuscript, approved final version of manuscript.

## FUNDING INFORMATION

The study was funded by Foundation for Scientific and Technological Development of Yugra, Grant Number 2022‐05‐02 (to ASG).

## CONFLICT OF INTEREST STATEMENT

The authors declare no conflict of interest.

## Data Availability

All data and code pertinent to the analysis presented in this paper are available at https://github.com/mrbarbitoff/alpinist_data_analysis/.
